# Exhaustion in tumor-infiltrating Mucosal-Associated Invariant T (MAIT) cells from colon cancer patients

**DOI:** 10.1007/s00262-021-02939-y

**Published:** 2021-04-22

**Authors:** William Rodin, Patrik Sundström, Filip Ahlmanner, Louis Szeponik, Kamil Kajetan Zajt, Yvonne Wettergren, Elinor Bexe Lindskog, Marianne Quiding Järbrink

**Affiliations:** 1 Dept of Microbiology and Immunology, Sahlgrenska Academy at the University of Gothenburg, Gothenburg, Sweden; 2 Dept of Surgery, Sahlgrenska Academy at the University of Gothenburg, Gothenburg, Sweden

**Keywords:** MAIT cell, Colorectal cancer, Exhaustion, PD-1, Tim-3, CD39

## Abstract

Mucosal-associated invariant T (MAIT) cells are unconventional T cells recognizing microbial metabolites, presented by the invariant MR1 protein. Upon activation, MAIT cells rapidly secrete cytokines and exert cytotoxic functions, and may thus be highly relevant also in tumor immunity. MAIT cells accumulate in colon tumors, but in contrast to other cytotoxic T cell subsets, their presence in tumors has been associated with worse patient outcome. Here we investigated if exhaustion may contribute to reduced anti-tumor immunity by MAIT cells. Freshly isolated lymphocytes from colon tumors, unaffected tissue and blood from the same patients were analyzed by flow cytometry to detect MAIT cells with effector functions that are relevant for tumor immunity, and their expression of inhibitory receptors and other exhaustion markers. Our studies show that MAIT cells with a PD-1^high^Tim-3^+^CD39^+^ terminally exhausted phenotype and an increased proliferation accumulate in colon tumors. The exhausted MAIT cells have reduced polyfunctionality with regard to production of important anti-tumor effector molecules, and blocking antibodies to PD-1 partly improved activation of tumor-infiltrating MAIT cells in vitro. We conclude that the tumor microenvironment leads to exhaustion not only of conventional T cells, but also MAIT cells, and that checkpoint blockade therapy may be useful also to reinvigorate tumor-infiltrating MAIT cells.

**Supplementary Information:**

The online version contains supplementary material available at 10.1007/s00262-021-02939-y.

## Introduction

Mucosal-associated invariant T (MAIT) cells are unconventional T cells typically expressing a semi-invariant T cell receptor (TCR) with V*α*7.2 joined to J*α*33, 20, or 12, which in turn is paired with a limited number of *β*-chains [1, 2]. Most MAIT cells are CD8^+^, but small populations of both CD4^+^ and double negative (DN) MAIT cells are also present in the circulation and peripheral tissues [2]. The MAIT TCR recognizes metabolites from the riboflavin synthesis pathway present in many, but not all, bacteria and fungi [3]. These ligands are presented by the invariant major histocompatibility complex class I-related protein 1 (MR1), which shows a remarkable conservation between species [4, 5]. MAIT cells can be found in most lymphoid and mucosal human tissues, but are most frequent in the liver [6]. In mucosal tissues, MAIT cells have a phenotype reminiscent of resident memory T cells [7–9], and they provide rapid responses to infection with bacteria and viruses. In particular, MAIT cells are programed for immediate cytokine production and cytotoxicity following activation, and both polyclonal and antigen-specific TCR-mediated stimulation results in production of IFN-γ, TNF, and IL-17 and up-regulation of intracellular Granzyme B (GrB). In addition, pro-inflammatory cytokines, for example the combination of IL-12 and IL-18, will also induce cytokine production in MAIT cells, independent of TCR signaling [10]. The propensity of MAIT cells to produce different cytokines further appears to be influenced by local tissue factors [11]. While circulating, intestinal, and hepatic MAIT cells preferentially produce TNF and IFN-γ, MAIT cells from infected lungs, the female genital tract, and breast epithelial ducts are much more prone to IL-17 and IL-22 production [8, 12–15].

The cytokine production and cytotoxicity of MAIT cells indicate that they may also contribute to immunity against tumors. It was recently demonstrated that MAIT cells accumulate in colon tumors, compared to the unaffected colon mucosa of the same individuals [14, 16, 17]. The tumor-infiltrating MAIT-cells produced IFN-γ and TNF, but only little IL-17. Their production of IFN-γ was, however, considerably lower than that of MAIT cells from the unaffected mucosa, while cytotoxic potential was similar in the two sites [14, 18]. In addition to the killing of tumor cells by CD8^+^ cytotoxic T cells, there is strong evidence that Th1 responses, and more specifically the balance between Th1 and Th17 responses, are important for patient outcome in colon cancer [19]. Therefore, MAIT cell contribution of IFN-γ may be an important part of anti-tumor defense, and the down-regulation of IFN-γ-production in tumor-associated MAIT cells may represent a tumor immune evasion mechanism. It should be noted, though, that MAIT cell enrichment in colon and hepatocellular carcinoma (HCC) correlates to a worse patient outcome [16, 20, 21].

Tumor-infiltrating conventional CD8^+^ T cells can enter a stage of exhaustion, due to sustained TCR activation, which is associated with reduced effector functions. This is a reversible state, and it is sustained by expression of inhibitory receptors such as programmed cell death protein 1 (PD-1), T cell immunoglobulin and mucin domain-3 (Tim-3), T cell immunoreceptor with Ig and ITIM domains (TIGIT), Lymphocyte activation gene 3 (LAG-3), and B and T lymphocyte attenuator (BTLA). T cell exhaustion was first defined in chronic viral infections [22], but has since been convincingly demonstrated also in tumors [23, 24].

Exhaustion has also been suggested in MAIT cells, mainly in the setting of chronic bacterial and viral infections. Generally, chronic infection or inflammation results in reduced frequencies of circulating MAIT cells, and the remaining MAIT cells display a prominent expression of PD-1 and reduced cytokine production. [7, 25–27]. Furthermore, the observation that blocking of Programmed death-ligand 1 (PD-L1)/PD-1 interactions in vitro partly restores IFN-γ-production in MAIT cells from tuberculosis patients further supports the notion of MAIT cell exhaustion [25]. Here, we investigated if tumor-infiltrating MAIT cells show signs of exhaustion, and if this would explain the poor cytokine responses in MAIT cells isolated from tumors. We could show that MAIT cells in colon tumors have an exhausted phenotype compared to MAIT cells from the unaffected colon mucosa and blood, express fewer effector molecules upon stimulation, and display partly increased activation following blocking of PD-1.

## Materials and methods

### Patients and tissue collection

Forty-seven individuals undergoing curative resection of colon tumors at the Sahlgrenska University Hospital were included in the studies (28 males and 19 females, aged 37 to 92, median age 74). Additional patient data is presented in Supplementary table S1. None of the patients suffered from autoimmune disease, were on immunomodulatory drugs, or had undergone radiotherapy or chemotherapy for at least three years prior to colectomy. Immediately after colectomy, a section of the tumor tissue encompassing both the center and more peripheral parts of the tumor was collected, as well as unaffected tissue from at least ten centimeters away from the tumor. The tissue material was transported in ice-cold PBS before isolation of lymphocytes within less than two hours. Heparinized venous blood was also obtained during surgery. Information about tumor stage was retrieved from the pathology report and medical records. Microsatellite instability (MSI), indicating the mutational load of the tumor, was analysed as previously described [18]. A tumor was defined as MSI high (MSI-H) if > 1 of 5 markers showed instability, and if no MSI was detected, the tumor was designated microsatellite stable (MSS).

### Cell isolation and stimulation

Lamina propria lymphocytes were isolated essentially as described [14]. Briefly, the tissue samples were washed with PBS and the muscle layers, fat, connective tissue and blood vessels were carefully removed. The tissue was cut into 5 mm pieces and subjected to four rounds of EDTA treatment to remove epithelial cells and intraepithelial lymphocytes. The remaining tissue was digested with Liberase TM (Roche) together with DNase I (Sigma Aldrich) for 2 h. The resulting single cell solution was re-suspended in RPMI 1640 (GIBCO® by Life Technologies™) containing 10% fetal bovine serum (Biological Industries), 25 mM of hepes, 100 U/ml of penicillin, 100 μg/ml of streptomycin, 292 μg/ml of L-glutamine (GIBCO™ Invitrogen Corporation), and 50 μg/ml of gentamicin (Lonza). Cells were manually counted with Tryphan blue to exclude dead cells. The isolation procedure resulted in a cell yield of 18 to 81 × 10^6^ mononuclear cells per gram unaffected tissue and 12 to 70 × 10^6^ cells per gram of tumor tissue. Viability of freshly isolated CD45^+^ cells from tissue was always above 85%. The enzymes employed for lymphocyte isolation did not affect detection of the markers used for MAIT cell identification, as parallel enzymatic treatment of PBMC did not reduce the surface expression of CD161 or V*α*7.2, or binding of the MR1 tetramer. PBMC were isolated by gradient centrifugation on Ficoll-Paque™Plus (GE Healthcare Bio-sciences AB).

To assess production of IFN-γ, GrB, IL-2, TNF, IL-17, and IL-22, cells were stimulated with 50 ng/mL of PMA and 500 ng/mL of ionomycin calcium salt (Sigma Aldrich) for 6 h, and a protein transport inhibitor (BD Golgi stop, BD Biosciences) was added 4 h before harvest of stimulated cells. In our experience, tissue-infiltrating MAIT cells sometimes express lower levels of TCR than circulating cells, and we thus used PMA and Ionomycin to circumvent the requirement for TCR engagement to activate MAIT cells in these experiments.

To determine the effect of checkpoint blockade on MAIT cell stimulation, single cell suspensions were stimulated with plate-bound antibodies to CD3 (coating over night with 100 µg/ml, clone: OKT3, Biolegend®), 100 µg/ml of soluble anti-CD28 (clone: CD28.2, Biolegend®), and 50 U/ml of recombinant IL-2 (R&D systems) for 48 h, in the presence or absence of 20 µg/ml of a blocking antibody to PD-1 (Pembrolizumab, Merck & Co. Inc.). Alternatively, PD-L2 expressing live THP-1 cells pre-incubated with *E. coli* [18] were used for MAIT cell stimulation for 24 h. MAIT cell expression of CD25, CD38 and HLA-DR was assessed by flow cytometry. As PD-1 primarily acts to reduce TCR-mediated signaling, we used polyclonal TCR and antigen-specific activation of MAIT cells in these experiments.

### Flow cytometry

Single cell suspensions were stained with CD4-FITC and -APC (clone OKT-4), CD14-FITC (clone M5E2), CD39-FITC (clone A1), TCR Vα7.2-APC and -BV421 (clone 3C10), PD-L2-PE-CF594 (clone 24F-10C12), and IL-22-APC (clone 2612A41) (all from Biolegend), CD3-Brilliant Violet 711 (clone UCHT1), CD3-APC-H7 (clone SK7), CD8-Brilliant Violet 711, -AF700, -BUV395 (clone RPA-T8), CD11c-BV421 (clone B-ly6), CD45-PerCP and -AF700 (clone 2D1), PD-1-BUV737 (clone EH21.1), Tim-3-BV650 (clone 7D3), BTLA-PE (clone J168-540), LAG-3-BV510 (clone T47-530), PD-L1-BV785 (clone MH1), Ki67-PE-Cy7 (clone B56), IFN-γ-BV421 (clone 4SB3), IL-2-PE (clone MQ1-17H12), IL-17-BV785 (clone N49-653), TNF-AF700 (clone MAB11), GrB-PE and -AF700 (clone GB11) (all from BD Biosciences™) and CD161-eFluor450 and -BV421 (clone HP-3G10) and TIGIT-PE (clone MBSA43) (eBioscience). APC- and PE-labeled tetramers of MR1 presenting the MAIT cell antigen 5-(2-oxopropylideneamino)-6-D-ribitylaminouracil (5-OP-RU) or the control antigen 6-formylpterin were kindly provided by the NIH tetramer core facility [3]. Lymphocytes were identified by their forward and side scatter characteristics combined with staining for CD45, and LIVE/DEAD Fixable Aqua Dead Cell Stain Kit (molecular probes® by Life Technologies™) was used to gate out dead cells. FIX&PERM® (AN DER GRUB Bio Research GmbH) intracellular staining kit was used for detection of cytokines, and True-Nuclear™ Transcription Factor Buffer Set (Biolegend®) for detection of Ki67. Flourescense minus one controls were used to determine positive staining. Data was acquired using Becton Dickinson LSR II and Fortessa X-20 flow cytometers and analyzed by FlowJo v10 software. A cut-off of minimum 100 events was applied in all analyses of cell surface markers and effector molecules. Polyfunctionality was evaluated using SPICE6™ software, and the polyfunctionality index calculated as previously described [28].

### Statistics

Statistical analyses of paired data were performed using two-tailed Wilcoxon matched-pairs signed rank test. When comparing three groups of matched data, the Friedman test followed by Dunn’s post test was used to achieve multiplicity adjusted P values. Correlations between age and MAIT cell characteristics and between expression of different exhaustion markers were performed using linear regression. Values of *p* < 0.05 were considered to be statistically significant.

## Results

### Identification of colonic MAIT cells

In order to investigate MAIT cells in colon tissues, we used freshly obtained tissue from colon cancer patients undergoing surgery, and isolated lymphocytes from the tumor, unaffected colon tissue and blood from the same patients. We and others have previously shown that the frequencies of MAIT cells are higher in the tumor tissue than the unaffected colon mucosa from the same individual [14, 16], and this finding was consistent also in this independent patient material (Supplementary Fig. S1a). When analyzing MAIT cell frequencies, we first used antibodies to V*α*7.2 combined with a high CD161 expression to identify MAIT cells within the CD45^+^CD3^+^ population. When MR1 tetramers loaded with the MAIT cell antigen 5-OP-RU became available these were used instead (see Supplementary Fig. S1b for gating strategies). MAIT cells can be CD8^+^, CD4^+^, or double negative (CD4^−^CD8^−^; DN). As CD4^+^ MAIT cells cannot be unambiguously identified by antibodies to V*α*7.2 and CD161 [29, 30], we restricted our analyses to CD8^+^ and DN MAIT cells.

In blood from healthy individuals, the large majority of MAIT cells express CD8, and this was also the case in colon cancer patients, both in blood and in the unaffected colon mucosa. However, CD8^+^ MAIT cells were less common in the tumor samples, which had more DN MAIT cells than any of the other tissues (Supplementary Fig. S1c). Previous studies have indicated that CD8^+^ and DN MAIT cells differ with regard to effector functions [29–31], and in the following experiments we therefore evaluated CD8^+^ and DN MAIT cells separately.

### The frequencies of MAIT cells with an exhausted phenotype are higher in tumors than in unaffected tissue.

**Fig. 1:**
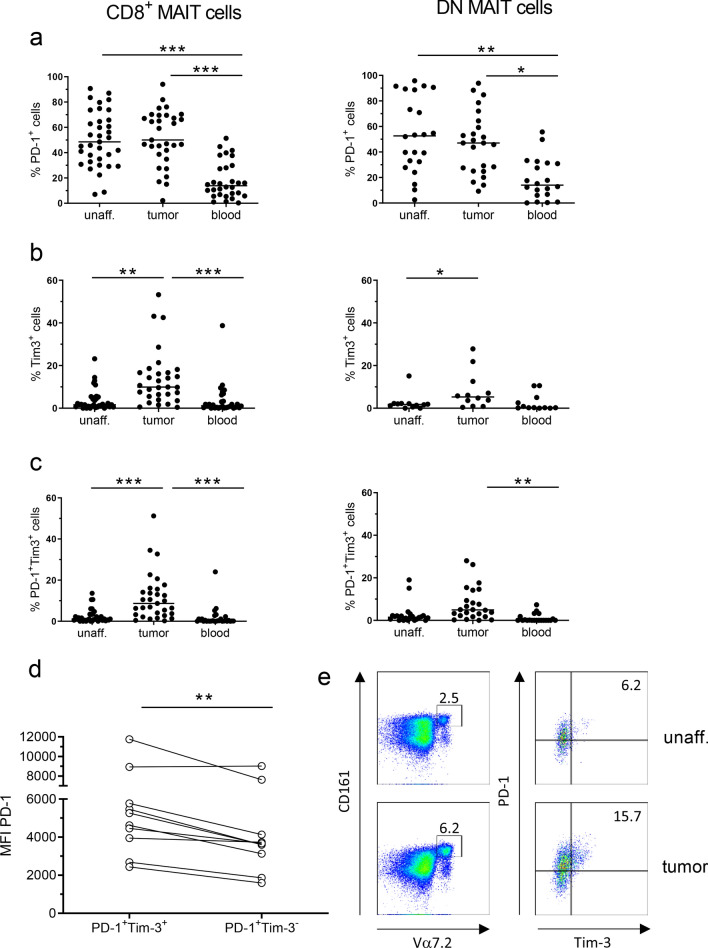
Expression of exhaustion markers on MAIT cells. Single cell suspensions were isolated from unaffected colon, colon tumors and peripheral blood, and CD8^+^ and DN MAIT cells analyzed for their expression of **a** PD-1, **b** Tim-3, or **c** co-expression of PD-1 and Tim-3 by flow cytometry. **d** MFI of PD-1 staining on Tim-3^+^ and Tim-3^−^ MAIT cells from colon tumors. **e** Representative flow cytometry plot showing Tim-3 and PD-1 expression on MAIT cells isolated from unaffected tissue and colon tumor from the same patient. Symbols represent individual values and the line the median. *p < 0.05, **p < 0.01, ***p < 0.001 in comparisons between different tissues using Wilcoxon matched-pairs signed rank test for paired values and the Friedman test followed by Dunn’s post test for multiple comparisons between tissues. *n* = 37

To examine if tumor-infiltrating MAIT cells have an exhausted phenotype, we analyzed their expression of the surface markers PD-1, Tim-3, CD39, TIGIT, BTLA, and LAG3, which have all been used to identify exhausted conventional CD8^+^ T cells in tumors [22, 32]. Flow cytometry analyses showed that there is no significant difference in frequencies of PD-1^+^ MAIT cells between tumors and unaffected tissue (Fig. 1a). Still, the mean fluorescence intensity (MFI) for PD-1 in the PD-1^+^ MAIT cell population was generally higher in cells from the tumor compared to the unaffected tissue (*p* < 0.01, Supplementary Fig. S2). About half of the mucosal MAIT cells expressed PD-1, while the expression was significantly lower in circulating MAIT cells (*p* < 0.01; Fig. 1a).

**Fig. 2:**
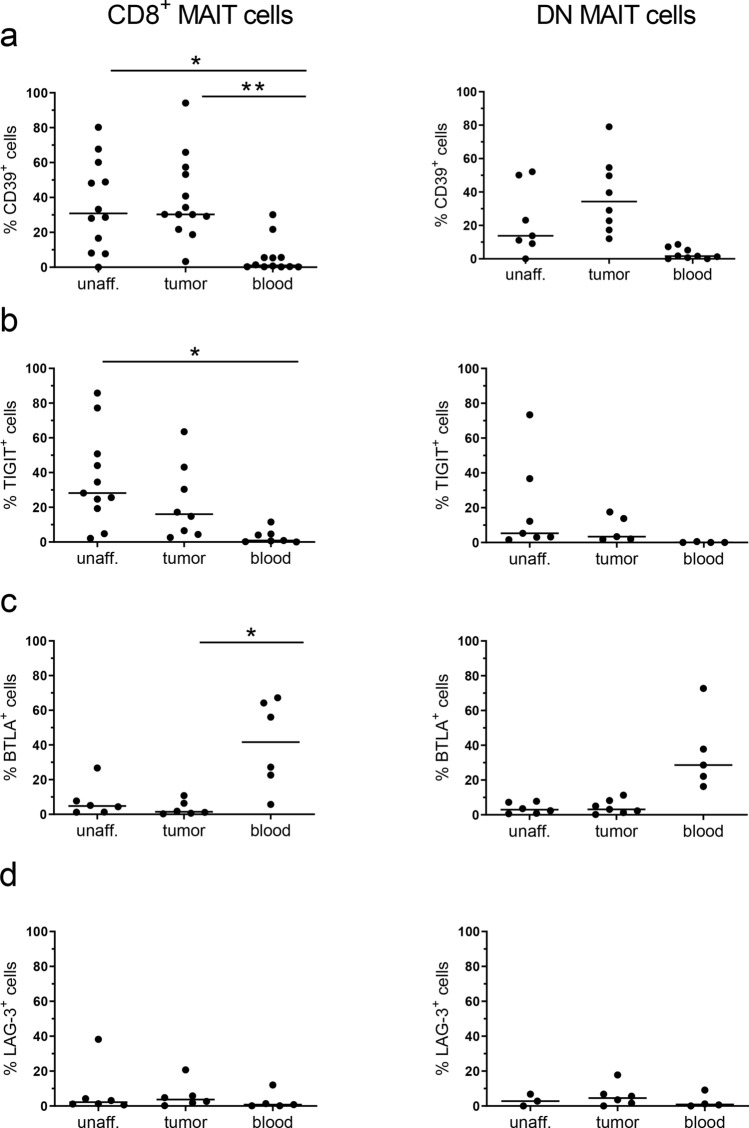
Expression of exhaustion markers on MAIT cells. Single cell suspensions were isolated from unaffected colon, colon tumors and peripheral blood, and CD8^+^ and DN MAIT cells analyzed for their expression of **a** CD39, **b** TIGIT, **c** BTLA, and **d** LAG-3 by flow cytometry. Symbols represent individual values and the line the median. *p < 0.05, **p < 0.01 in comparisons between different tissues using the Friedman test followed by Dunn’s post test for multiple comparisons. *n* = 6–13

When we extended the analysis to comprise other exhaustion markers, we could see a substantial increase in the frequencies of MAIT cells expressing Tim-3 in the tumors compared to the unaffected tissue (*p* < 0.01; Fig. 1b). Most of these Tim-3^+^ MAIT cells in the tumors also expressed PD-1 (Fig. 1c), and it was also clear that the Tim-3^+^ MAIT cells in the tissue had a brighter PD-1 staining than the PD-1^+^Tim-3^−^ cells (*p* < 0.01; Fig. 1d, e). Co-expression of PD-1 and Tim-3 marks severely exhausted conventional T cells in chronic viral infections and tumors [33, 34], and we thus considered the PD-1^high^Tim-3^+^ MAIT cells as the most exhausted subset. We also examined the expression of additional inhibitory receptors on tumor-infiltrating MAIT cells, and found that although the frequencies of CD39^+^ and TIGIT^+^ MAIT cells varied between individuals, they were generally higher in mucosal than in circulating MAIT cells, especially in the CD8^+^ MAIT cells (*p* < 0.05–0.01; Fig. 2a, b). In contrast, only about 10% of MAIT cells in both tumors and unaffected mucosa expressed BTLA and LAG3 (Fig. 2c, d), while many more circulating MAIT cells were BTLA^+^. However, there was no significant difference in expression of any of these exhaustion markers between tumors and the corresponding unaffected tissues.

**Fig. 3:**
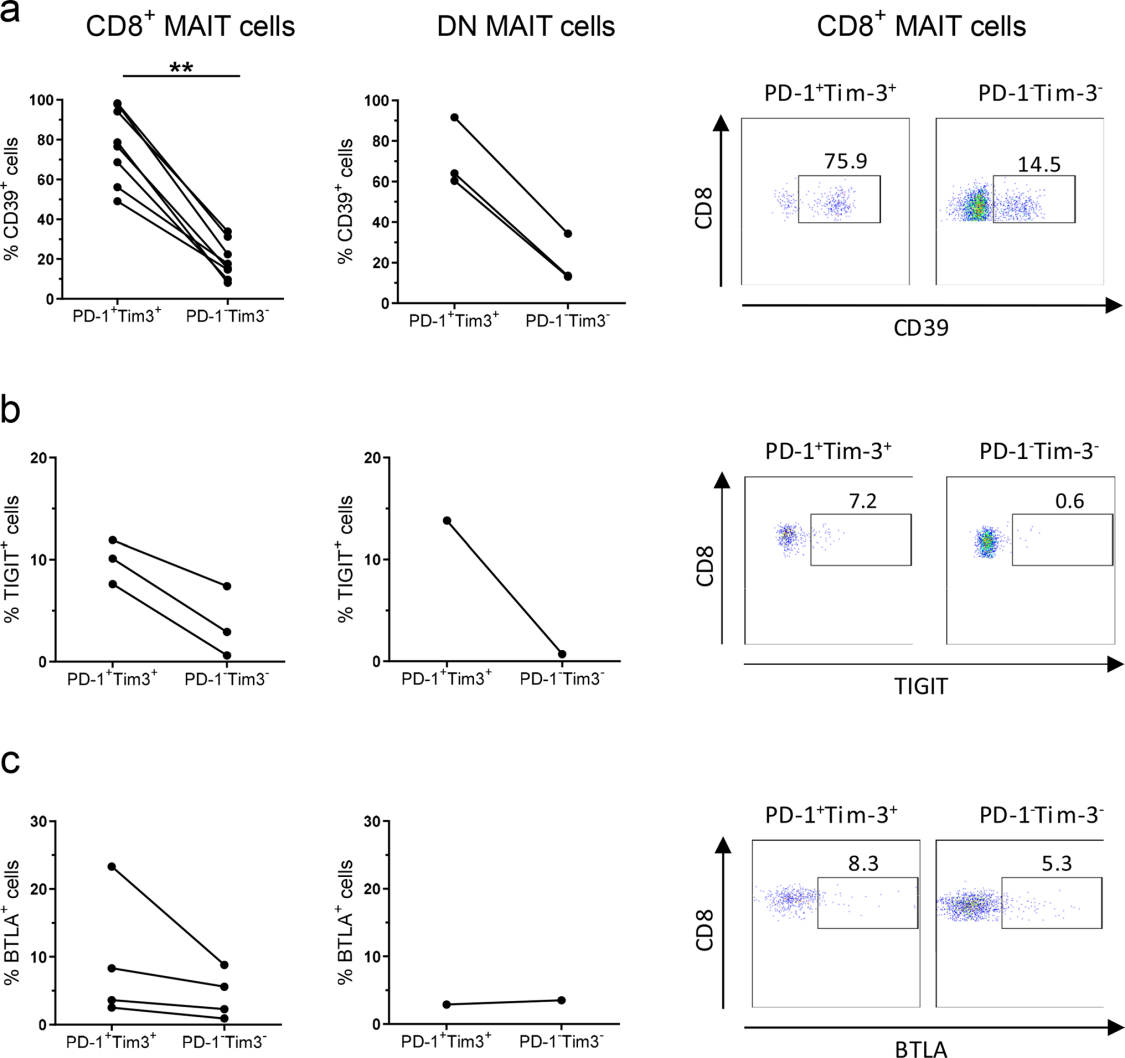
Expression of exhaustion markers on MAIT cells. Single cell suspensions were isolated from colon tumors, and CD8^+^ and DN MAIT cells further sub-divided into PD-1^high^Tim-3^+^ and PD-1^−^Tim-3^−^ populations. These two populations were analyzed for their expression of **a** CD39, **b** TIGIT, and **c** BTLA by flow cytometry. Symbols represent individual values, and are connected to show corresponding values in the same individuals. **p < 0.01 using Wilcoxon matched-pairs signed rank test. *n* = 3–8

Expression of multiple inhibitory receptors is a cardinal feature of exhausted cells [22], and we therefore analyzed the co-expression of CD39, TIGIT, and BTLA on the PD-1^high^Tim-3^+^ putatively exhausted MAIT cells. These analyses could only be carried out in the tumors, and not in all individuals, as cell numbers were otherwise too low to obtain reliable data. Multicolor flow cytometry analyses clearly showed that the PD-1^high^Tim-3^+^ MAIT cells also expressed CD39 to a large extent, regardless of CD8 expression, and much more than the PD-1^−^Tim-3^−^ MAIT cells (Fig. 3a). Such a difference was not recorded for TIGIT or BTLA, although their expression was somewhat more prominent in the PD-1^high^Tim-3^+^ MAIT cells (Fig. 3b, c). These results were also confirmed in analyses of correlation between surface markers in all CD8^+^ MAIT cells in the tumors. Tim-3 expression correlated positively with PD-1 and CD39 expression (*p* < 0.001; Supplementary Fig. S3a, b), but not with TIGIT or BTLA expression. In contrast, PD-1 expression did not correlate to any other marker than Tim-3. In the unaffected tissue, no corresponding correlation could be documented.

**Fig. 4:**
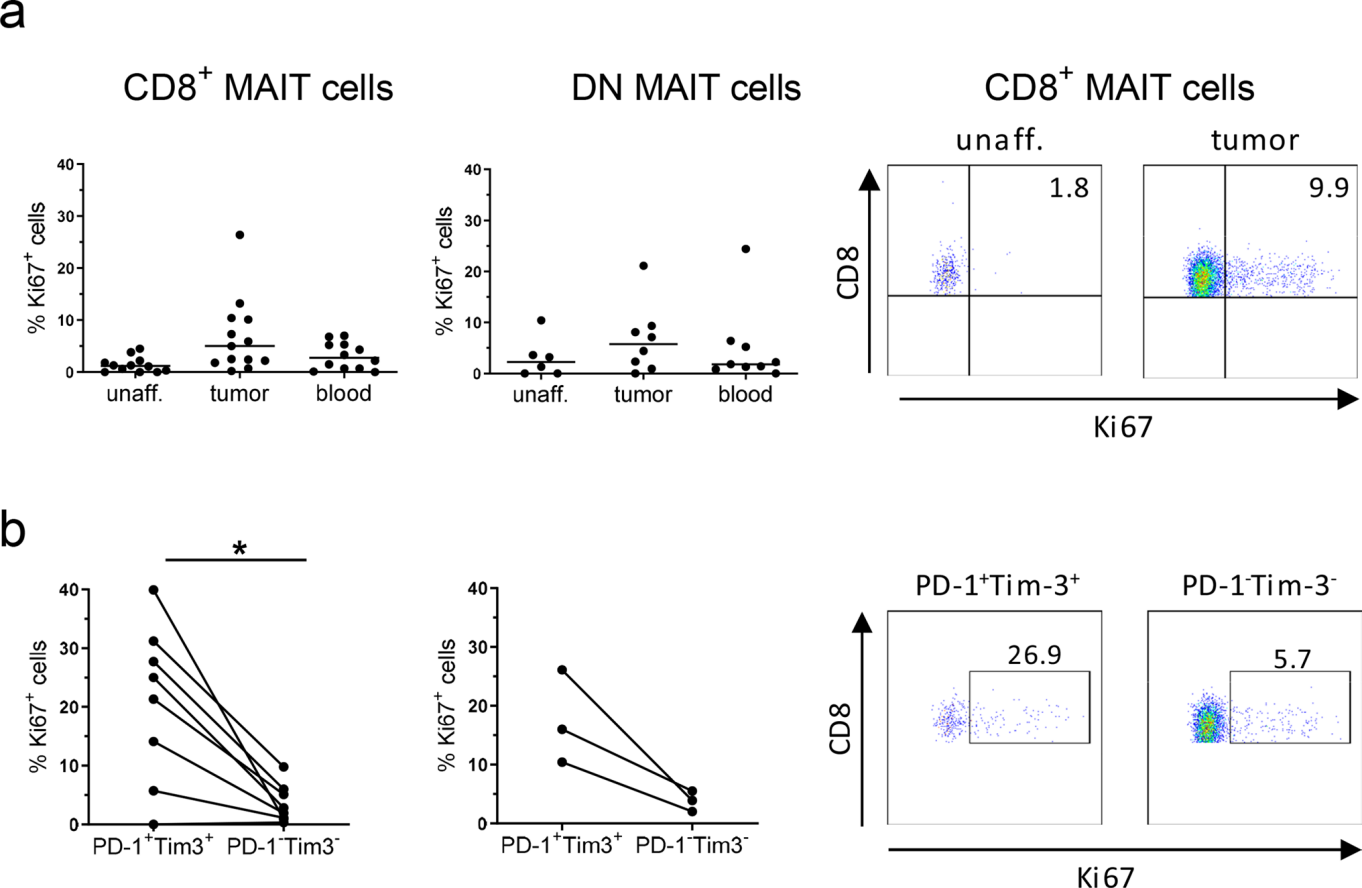
Proliferation of MAIT cells. Single cell suspensions were isolated from unaffected colon, colon tumors and peripheral blood, and CD8^+^ and DN MAIT cells analyzed for their expression of Ki67 by flow cytometry (**a**). **b** CD8^+^ and DN MAIT cells from colon tumors were further sub-divided into PD-1^high^Tim-3^+^ and PD-1^−^Tim-3^−^ populations. These two populations were then analyzed for their expression of Ki67. Symbols represent individual values and the line the median. In **b**, symbols are connected to show corresponding values in the same individuals. *p < 0.05 using Wilcoxon matched-pairs signed rank test for paired values. *n* = 13

We also examined ongoing proliferation in the MAIT cell subsets, by assessing Ki67 expression ex vivo, and found that tumor-infiltrating CD8^+^ MAIT cells proliferated somewhat more than those in the unaffected tissue or circulation (Fig. 4a). When looking specifically at PD-1^high^Tim-3^+^ MAIT cells, they had higher expression of Ki67 than their PD-1^−^Tim-3^−^ counterparts in the tumors (*p* < 0.05), regardless of CD8 expression (Fig. 4b). Linear regression also showed that Tim-3 expression correlated to Ki67 expression in CD8^+^ MAIT cells from the tumors (*p* < 0.001; Supplementary Fig. S3c).

To assess if MAIT cell exhaustion was dependent on tumor stage or microsatellite status, we correlated these factors to the frequencies of PD-1^high^Tim-3^+^ cells in the CD8^+^ MAIT cells in the tumors. These analyses revealed no significant associations between accumulation of PD-1^high^Tim-3^+^ MAIT cells and tumor stage (I-IV) or tumor invasion (T2-T4). Furthermore, exhaustion in tumor-infiltrating MAIT cells was not correlated to patient age or microsatellite status (Supplementary Fig. S4).

Taken together, these observations demonstrate that MAIT cells with an exhausted PD-1^high^Tim-3^+^CD39^+^ phenotype and increased proliferation accumulate in the tumor microenvironment. In addition, circulating and tissue-resident MAIT cells have a distinctly different expression of exhaustion markers.

### Exhausted tumor-infiltrating MAIT cells have reduced effector functions

In viral infections, exhaustion of conventional T cells results in a loss of effector functions [22, 32]. We previously described a reduction in the frequencies of IFN-γ-producing MAIT cells in colon tumors [14], and we now wanted to examine if MAIT exhaustion contributed to reduced cytokine production in tumor-infiltrating MAIT cells. We therefore assessed the production of several effector molecules important for T cell mediated tumor immunity (IFN-γ, GrB, IL-2, IL-17, IL-22, TNF) following polyclonal stimulation with PMA and Ionomycin. When all MAIT cells were analyzed together, IFN-γ-production was lower in MAIT cells from the tumors than in cells from the unaffected colon (median 33% in tumor-derived and 50% in unaffected colon MAIT cells, *p* < 0.05), while there were no significant differences in total MAIT cell production of GrB (median 46% *vs* 28%), IL-2 (median 26% *vs* 21%), or TNF (median 61% *vs* 41%) between cells from tumors and unaffected tissue. In contrast to these cytokines, IL-17-producing MAIT cells (median 0.1% in tumor-derived *vs* 0.4% in unaffected colon MAIT cells) and IL-22-producing MAIT cells (median 0.5% *vs* 0.6%) were scarce in both tissues.

**Fig. 5:**
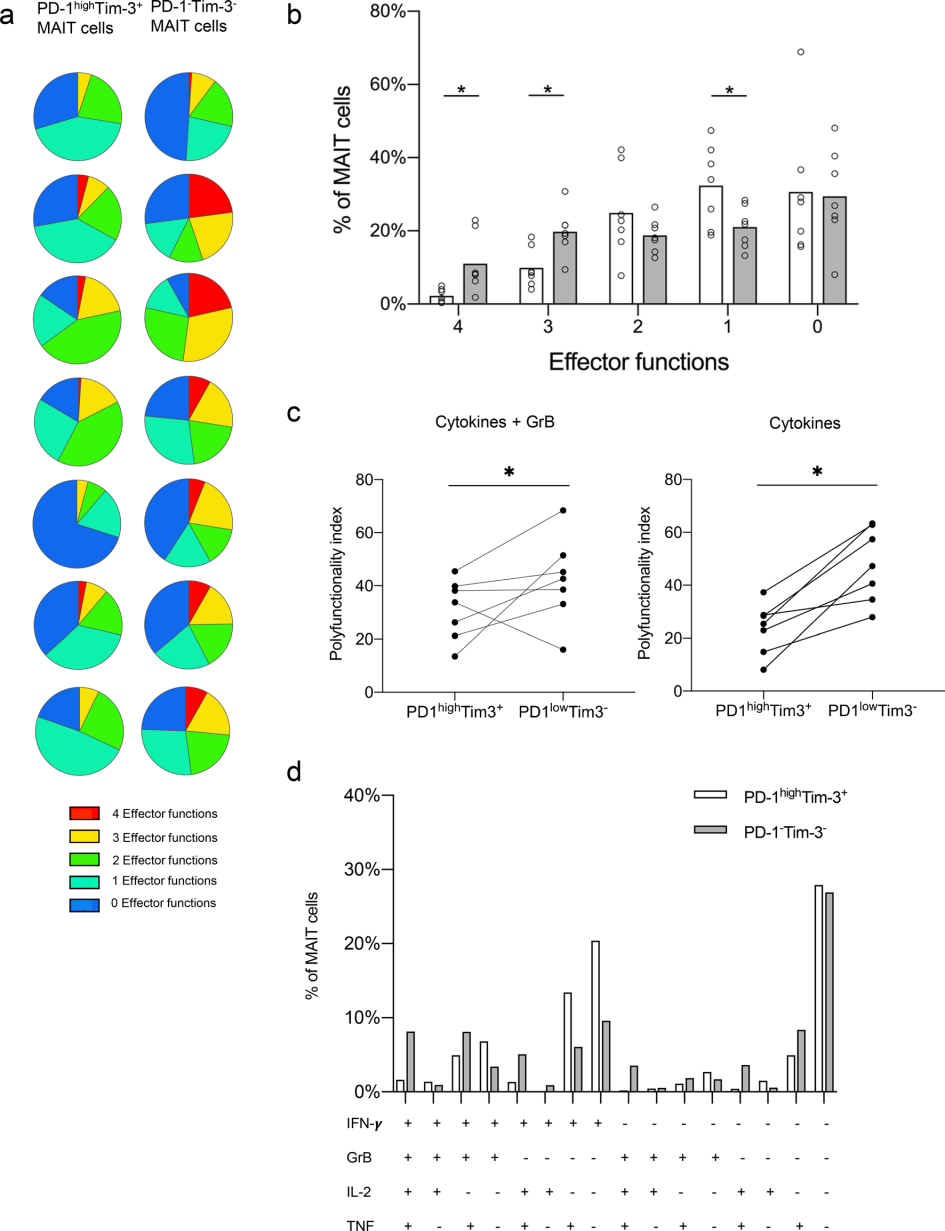
Polyfunctionality in tumor-infiltrating MAIT cells. Single cell suspensions were isolated from colon tumors, and production of IFN-γ, GrB, IL-2, and TNF by MAIT cells evaluated in vitro by flow cytometry after polyclonal stimulation with PMA and Ionomycin. **a** Percentage of PD-1^hi^Tim-3^+^ and PD-1^−^Tim-3^−^ MAIT cells from individual patients expressing 4 (red), 3 (yellow), 2 (green), 1 (turquoise) or 0 (blue) of the analyzed effector molecules illustrated as pie charts. **b** Frequencies of PD-1^hi^Tim 3^+^ (white bars) and PD-1^−^Tim 3^−^ (grey bars) MAIT cells expressing 4, 3, 2, 1, or 0 of the analyzed effector molecules, shown as the percentage of total MAIT cells. **c** Polyfunctionality index for PD-1^hi^Tim-3^+^ and PD-1^−^Tim-3^−^ MAIT cells analyzed for 4 (GrB and cytokines) or 3 effector molecules (only cytokines). **d** Frequencies of PD-1^hi^Tim-3^+^ (white bars) and PD-1^−^Tim-3^−^ (grey bars) MAIT cell subsets expressing a combination of IFN-γ, GrB, IL-2, and TNF shown as the percentage of total MAIT cells. Symbols represent individual values and bars the mean. *p < 0.05 using Wilcoxon matched-pairs signed rank test. *n* = 7

TCR-mediated stimulation of MAIT cells results in a substantial up-regulation of PD-1 and Tim-3 on the cell surface (1.4-fold increase in PD-1^+^ and 2.8-fold increase in Tim-3^+^ tumor-derived MAIT cells). We therefore used short-term stimulation with PMA/Ionomycin, which does not change the expression of PD-1 and Tim-3 on MAIT cells, to be able to detect cytokine responses in cells with an exhausted phenotype. When cytokine production was analyzed in the context of exhaustion markers, multiparameter analyses showed that the PD-1^high^Tim-3^+^ MAIT cell population in the tumors had a significant loss of cells with multiple functions, i.e. cells expressing all four or all but one of the investigated effector molecules, when compared to PD-1^−^Tim-3^−^ MAIT cells from the same tumor (*p* < 0.05; Fig. 5a,b). This feature was also demonstrated by a reduced polyfunctionality index in PD-1^high^Tim-3^+^ MAIT cells from the tumors compared to the PD-1^−^Tim-3^−^ MAIT cells (Fig. 5c). To determine the extent of the GrB contribution to the reduced polyfunctionality, the analysis was repeated with only the three cytokines. These calculations showed that the difference in polyfunctionality between PD-1^high^Tim-3^+^ and PD-1^−^Tim-3^−^ MAIT cells was even more pronounced without GrB (Fig. 5c and Supplementary Fig. S5), indicating that there is no specific weakening of the cytolytic capacity in the exhausted MAIT cells. When individual combinations of the different effector molecules were investigated, the largest loss in the exhausted MAIT cells was seen in cells expressing all four effector molecules or all but GrB and/or IFNγ (Fig. 5d). The few IL-17- and IL-22-producing cells present in the tumors were analysed separately, and there was no evidence of enrichment in either the PD-1^high^Tim-3^+^ or PD-1^−^Tim-3^−^ MAIT cell fractions (Data not shown). MAIT cells in the unaffected tissue and in blood also displayed reduced effector functions in PD-1^high^Tim-3^+^ cells, although not to the same extent as the tumor-associated MAIT cells (Data not shown).

### MAIT cell activation can be restored by blocking PD-1

**Fig. 6:**
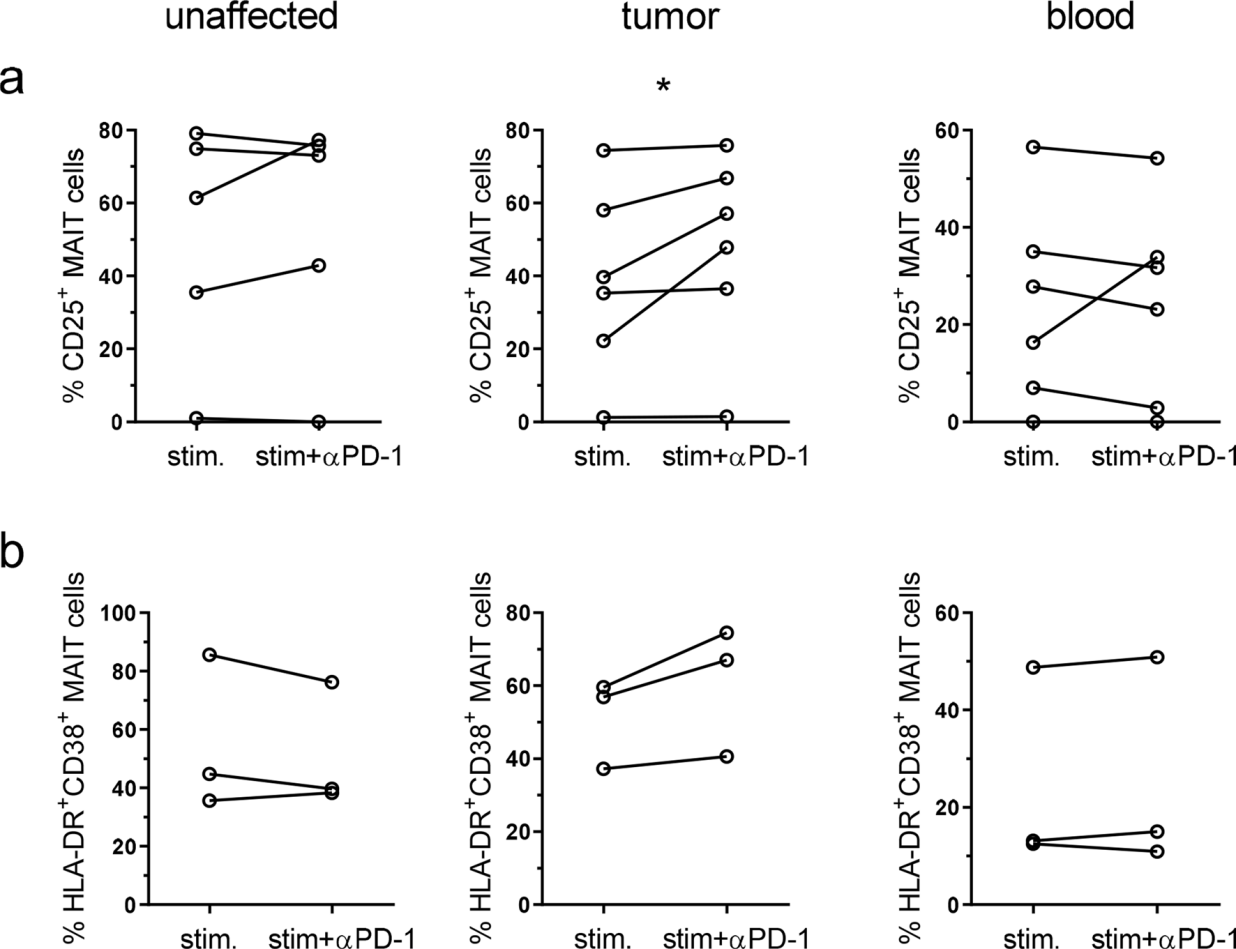
MAIT cell activation after PD-1 blocking. Single cell suspensions were isolated from unaffected colon, colon tumors and peripheral blood, and stimulated with anti-CD3, anti-CD28 and IL-2 (**a**) or THP-1 cells pre-incubated with *E. coli* (**b**) in the presence or absence of blocking antibodies to PD-1. MAIT cell expression of CD25 (**a**) or co-expression of HLA-DR and CD38 (**b**) was evaluated by flow cytometry. Symbols represent individual values, and are connected to show corresponding values in the same individuals. *p < 0.05 using Wilcoxon matched-pairs signed rank test. *n* = 3–6

PD-1 interaction with the ligands PD-L1 and PD-L2 reduces TCR-mediated signaling in conventional T cells, but signaling can be restored by blocking antibodies to either PD-1 or the ligands [23, 24]. The single cell suspensions from tumors contain several subsets of PD-L1 and PD-L2 expressing cells, primarily monocytes/macrophages and dendritic cells (See Supplementary Fig. S6 for representative FACS plots). To investigate if these ligands may reduce activation in exhausted tumor-infiltrating MAIT cells, and thus if MAIT cells might respond to checkpoint blockade therapy, we stimulated MAIT cells with anti-CD3 and anti-CD28 antibodies in the presence or absence of neutralizing antibodies to PD-1. These experiments were performed with the bulk lamina propria cell fractions, to try to mimic many of the signals a tissue-resident MAIT cell would receive in vivo. Tumor-infiltrating MAIT cells were readily stimulated by anti-CD3/CD28 treatment to express CD25. In unstimulated MAIT cells 3.8% (median) expressed CD25 in the unaffected tissue, 5.1% in the tumors, and 0% in the blood, and CD25 expression was strongly increased in all the tissues following activation (Fig. 6a). Stimulation also strongly increased the expression of PD-1 on MAIT cells from all three locations. The presence of blocking antibodies to PD-1 significantly increased expression of CD25 on tumor-infiltrating MAIT cells (*p* < 0.05; Fig. 6a). The effect of the in vitro checkpoint blockade treatment varied between individuals, but in 3 out of 6 patients a reasonable (> 15%) increase could be detected on MAIT cells from the tumors. In contrast, MAIT cells from unaffected tissue or blood did not increase their expression of CD25 to the same extent following PD-1 blockade. The responses seen were not correlated to MAIT cell expression of PD-1 before stimulation, or tumor stage or microsatellite status. In a second set of experiments, we used live THP-1 cells presenting antigens from *E. coli* to stimulate tissue-derived MAIT cells. This stimulation also increased CD25 on MAIT cells, but presence of blocking antibodies to PD-1 did not change frequencies of CD25^+^ MAIT cells (Data not shown). In this experimental system, we also assessed co-expression of HLA-DR and CD38, as a second means to evaluate MAIT cell activation. The co-expression of HLA-DR and CD38 on MAIT cells was increased upon stimulation, and a further increase was seen in two out of the three patients available for examination, if PD-1 was blocked during activation (Fig. 6b). The effect was mainly a result of increased CD38 expression when PD-1 was blocked, as HLA-DR was already high following stimulation with antigen-presenting THP-1 cells (Supplementary Fig. 7). As with the anti-CD3 and anti-CD28 stimulation, the effect of anti-PD-1 antibodies was only seen in the tumor-infiltrating MAIT cells. Taken together, these analyses show that PD-1 blockade can improve activation in tumor-infiltrating MAIT cells in a subset of colon cancer patients.

## Discussion

In this study, we show that the MAIT cells accumulating in human colon tumors differ phenotypically from those in the unaffected mucosa and blood in several ways. Tumor-infiltrating MAIT cells have a different distribution of CD8^+^ and DN MAIT cell subsets and display surface markers indicating an exhausted state. Furthermore, the PD-1^high^Tim-3^+^ phenotypically exhausted MAIT cells have reduced effector functions, but the presence of blocking antibodies to PD-1 increase their activation.

MAIT cells have generally been described as CD8^+^ or DN, while CD4^+^ MAIT cells usually make up a minute population, at least in the blood and liver [2, 20, 29–31]. In the current study, the DN MAIT cells were enriched in the tumors compared to both unaffected tissues and blood*.* It has been suggested that DN MAIT cells develop from CD8^+^ MAIT cells and that they represent a more mature MAIT cell population [29]. TCR-mediated activation in vitro results in a profound loss of CD8 and subsequent apoptosis in MAIT cells [29], and this process may be accelerated in the tumor microenvironment, where an impaired epithelial barrier allows influx of bacterial metabolites [35, 36]. DN MAIT cells in the circulation express less cytotoxic markers and Th1-associated cytokines than CD8^+^ MAIT cells, and are more prone to apoptosis. There is also an increased production of IL-17 from DN MAIT cells, albeit from a low starting level [29, 31]. Also in the rectal mucosa, CD8^+^ MAIT cells appear to have more pro-inflammatory functions than DN cells [37]. If these features of DN MAIT cells are consistent also in the tumor microenvironment, DN MAIT cells may be less functional with regard to anti-tumor immunity, and their increased presence may partly explain the reduced cytokine responses recorded in tumor-infiltrating MAIT cells [14, 20, 38].

To further evaluate the effect of the tumor microenvironment on MAIT cell effector functions, we investigated the presence of exhausted MAIT cells in the tumors. Exhaustion was originally described as a sequential, but reversible, loss of effector functions due to consistent antigen stimulation and inflammation in mice chronically infected with lymphocytic choriomeningitis virus [22]. Exhaustion has since become evident in many infections, and also in tumors, and is sustained by expression of inhibitory receptors, and also involves changes in transcription factor expression and proliferative capacity [22, 32]. Our analyses showed a markedly increased frequency of PD-1^high^Tim-3^+^ exhausted MAIT cells in the tumors compared to both unaffected colon tissue and blood. Further analyses showed that PD-1^high^Tim-3^+^ cells also co-express CD39, and that they have an increased proliferation. Tumor cells can exhibit a prominent expression of the PD-1 ligands PD-L1 and PD-L2, and also produce the Tim-3 ligand Galectin-9, and may thus directly contribute to maintain the exhausted state of infiltrating T cells. In colon tumors, however, infiltrating immune cells appears to be a major source of PD-L1 and PD-L2, as well as Galectin-9 [39, 40], and may thus modify MAIT cell function in human colorectal cancer. It is also possible that additional factors from cancer cells contribute to reduced effector functions in MAIT cells [14, 20]. A recent study indicates that bacteria present in colorectal tumors induce CD39 expression following TCR-mediated stimulation [36], and chronic bacterial stimulation may thus contribute to MAIT cell exhaustion in the tumor microenvironment. CD39 is an ectoenzyme converting pro-inflammatory ATP to immunosuppressive adenosine which is expressed on Treg and exhausted conventional CD8^+^ T cells [41], and this way exhausted MAIT cells may contribute directly to reducing anti-tumor immunity. Whether MAIT cells themselves are sensitive to adenosine-mediated immunosuppression is not yet known, but presents an intriguing possibility of negative autocrine regulation of exhausted MAIT cells.

Another important observation is that colonic MAIT cells were distinctly different from circulating cells, with regard to expression of most of the exhaustion markers analyzed. These inhibitory receptors were generally higher in the tissue than in the circulation, with the exception of BTLA, which had a reversed expression. Similar results were recently also shown for PD-1, TIGIT, and LAG-3 by Schmaler et al., who compared MAIT cells from normal colon mucosa and blood [42]. These observations underline the importance of using MAIT cells from the organ of interest, and not extrapolating from circulating cells, even though they may be isolated from a relevant patient population.

Previous studies have suggested MAIT cell exhaustion in chronic bacterial and viral infections [7, 25, 26], and more recently, also in HCC and colorectal cancer [20, 36]. In HCC, MAIT cells express more PD-1 and Tim-3 than in the surrounding liver tissue, even though expression levels were generally much lower than in colon mucosa and tumors, respectively [20]. Interestingly, co-culture with HCC cell lines resulted in increased PD-1 and Tim-3 expression on MAIT cells. MAIT cell exhaustion in the tumor microenvironment may thus be induced in parallel to, or shortly after, strong stimulation, as previously demonstrated for bacterial infections [43]. In the current study, we could also correlate the exhausted MAIT cell phenotype with reduced functional capacity, as PD-1^high^Tim-3^+^ tumor-infiltrating MAIT cells have reduced effector polyfunctionality. Our studies show no selective loss of one certain cytokine from exhausted MAIT cells, but rather a decline in polyfunctionality. However, GrB appears to be kept in the phenotypically exhausted MAIT cells to a larger extent than IL-2, TNF, and IFN-γ, and did not contribute to the reduced polyfunctionality. Likewise, it has previously been shown that exhausted conventional CD8^+^ T cells still express high levels of GrB, and retain their cytotoxic ability [22, 32]. This finding is compatible with our recent observation that MAIT cells from tumors and unaffected tissues have comparable cytotoxic potential [18].

Recently, it has become clear that the pool of exhausted CD8^+^ cells present in chronic viral infection and in tumors can be further divided into progenitor exhausted cells and terminally exhausted cells. Progenitor exhausted cells are distinguished by an intermediate expression of PD-1, CXCR5, and the transcription factor Tcf1, and upon TCR-mediated stimulation they can differentiate into terminally exhausted cell co-expressing high levels of PD-1 together with Tim-3 and CD39 [41, 44, 45]. The terminally exhausted cells are still highly cytotoxic and proliferate more in the tumor microenvironment, but have reduced long-term survival and reduced polyfunctionality with regard to cytokine production [44, 46]. The PD-1^high^Tim-3^+^CD39^+^ phenotype, increased proliferation, and reduced polyfunctionality of tumor-infiltrating MAIT cells all suggest that they are terminally exhausted. It has been shown in murine studies that only progenitor exhausted T cells can respond to anti-PD-1 immunotherapy [44, 45], and in human tumors, the presence of progenitor exhausted cells correlates to a better response to anti-PD-1 therapy [44, 47]. However, presence of PD-1^high^ cells with a phenotype reminiscent of terminally exhausted cells in non-small cell lung cancer also strongly correlated to treatment response with anti-PD-1 [48]. Nevertheless, it is not clear if these observations apply to unconventional T cells like MAIT cells. In this study, we show that the presence of PD-1 blocking antibodies during in vitro stimulation increases activation of tumor-infiltrating MAIT cells in a subset of colon cancer patients, indicating that checkpoint blockade may act on unconventional T cells from cancer patients. A recent study showed that both PD-1 and Tim-3 can bind to Galectin-9 in vitro, and that simultaneous PD-1 signaling actually prevents apoptosis induced by the Galectin-9 Tim-3 axis [49]. Therefore, combining blocking of PD-1 and galectin-9 might give a further mechanistic insight into the regulation of MAIT cell responsiveness. We cannot say if the reinvigorated cells responding to checkpoint therapy are only the terminally exhausted cells retrieved from the tumors, as the stimulation protocol leads to up-regulation of PD-1 in the majority of MAIT cells. Furthermore, the effect of PD-1 blocking may be underestimated in our experimental system, as there are probably substantial amounts of potentially MAIT-stimulating cytokines induced by the polyclonal stimulation or presence of bacteria. The effect of checkpoint blockade is probably weaker if the MAIT cell is stimulated by mixed cytokine and TCR signals compared to TCR-mediated signaling alone.

In the few studies available to date, MAIT cell infiltration into tumors is associated with reduced effector functions and impaired patient outcome [14, 16, 20, 21]. This may partly be caused by an accumulation of exhausted MAIT cells in tumors, but our results suggest that immune checkpoint blockade may improve MAIT cell function, and may thus contribute to improved anti-tumor immunity in the tumor microenvironment also from resident MAIT cells. It is also encouraging to note that terminally exhausted conventional CD8^+^ T cells retain efficient cytolytic activity and can kill tumor cells in vitro and reduce tumor growth in vivo [44]. Taken together, our results indicate that the tumor microenvironment inflict a state of exhaustion in tumor-infiltrating MAIT cells, and that checkpoint blockade might reinvigorate MAIT cells to increase anti-tumor effector functions.

## Conclusions

In conclusion, our study shows that MAIT cells infiltrating colon tumors have a terminally exhausted phenotype and increased proliferation. They also show reduced polyfunctionality with regard to effector molecules important for anti-tumor immunity, another hallmark of exhausted conventional CD8^+^ T cells. Furthermore, in vitro immune checkpoint blockade with anti-PD-1 antibodies improved activation in tumor-infiltrating MAIT cells. These results indicate that MAIT cells are exhausted by the tumor microenvironment in colon tumors, and that checkpoint blockade therapy may be useful to unleash anti-tumor effector functions in MAIT cells, a feature that may contribute to the success of these therapies.

### Supplementary Information

Below is the link to the electronic supplementary material.

Supplementary file1 (PDF 983 kb)

Supplementary file2 (PDF 104 kb)

## Abbreviations

BTLA: B and T lymphocyte attenuator
DN: Double negative
GrB: Granzyme B
HCC: Hepatocellular carcinoma
LAG-3: Lymphocyte activation gene 3
MAIT cell: Mucosal-associated invariant T cell
MFI: Mean fluorescence intensity
MR1: Major histocompatibility complex class I-related protein 1
MSI-H: Microsatellite instability high
MSS: Microsatellite stable
PD-1: Programmed cell death protein 1
PD-L1: Programmed death-ligand 1
PD-L2: Programmed death-ligand 2
TCR: T cell receptor
TIGIT: T cell immunoreceptor with Ig and ITIM domains
Tim-3: T cell immunoglobulin and mucin domain-3
5-OP-RU: 5-(2-Oxopropylideneamino)-6-D-ribitylaminouracil
