# Supplementary material for: Exhaustion in tumor-infiltrating Mucosal-Associated Invariant T (MAIT) cells from colon cancer patients
Source: Cancer Immunol Immunother. 2021 Apr 22;70(12):3461–75. doi: 10.1007/s00262-021-02939-y (PMC8571139; doi:10.1007/s00262-021-02939-y)
Supplement: Supplementary file 1 — Supplementary file1 (PDF 983 kb) [file 262_2021_2939_MOESM1_ESM.pdf]

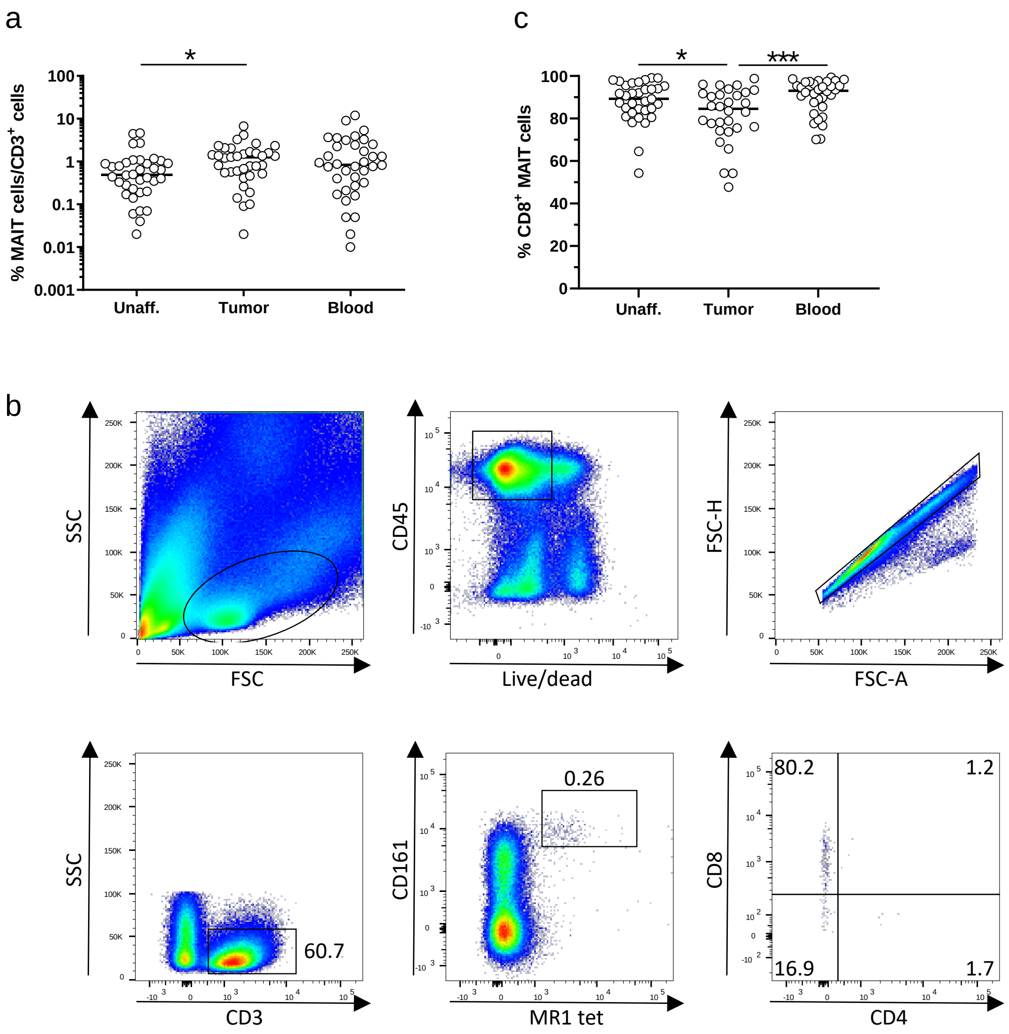

Supplementary Fig. S1 Gating strategy to identify MAIT cells. (a) Single cell suspensions were isolated from unaffected colon, colon tumors and peripheral blood, and frequencies of MAIT cells among $CD3^+$ cells determined by flow cytometry. (b) Dot plots show one example of MAIT cell gating using a single cell suspension from a colon tumor. Live, singlet cells from a lymphocyte gate were further gated as $CD45^+CD3^+$ T cells, and then Vα7.2 and CD161 or MR1 tetramers were used to identify MAIT cells. (c) Distribution of $CD8^+$ and DN MAIT cells in cell suspensions from unaffected colon, colon tumors and peripheral blood. Symbols represent individual values. * $p<0.05$, *** $p<0.001$, using the Friedman test followed by Dunn's post test for multiple comparisons. n=41

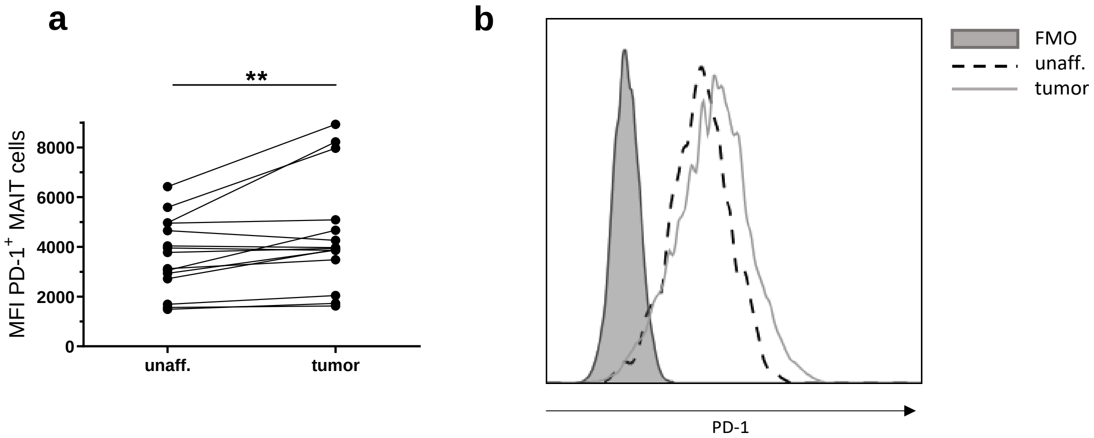

Supplementary Fig. S2 PD-1 expression in tissue MAIT cells. Single cell suspensions were isolated from unaffected colon and colon tumors, and MAIT cells were analyzed for their expression of PD-1 by flow cytometry. (a) Mean fluorescence intensity (MFI) of PD-1 staining on MAIT cells from unaffected colon and colon tumors. (b) Representative histogram showing PD-1 expression on MAIT cells isolated from a colon tumor and the corresponding unaffected tissue. Symbols represent individual values and the line the median. ** $p<0.01$, n=15

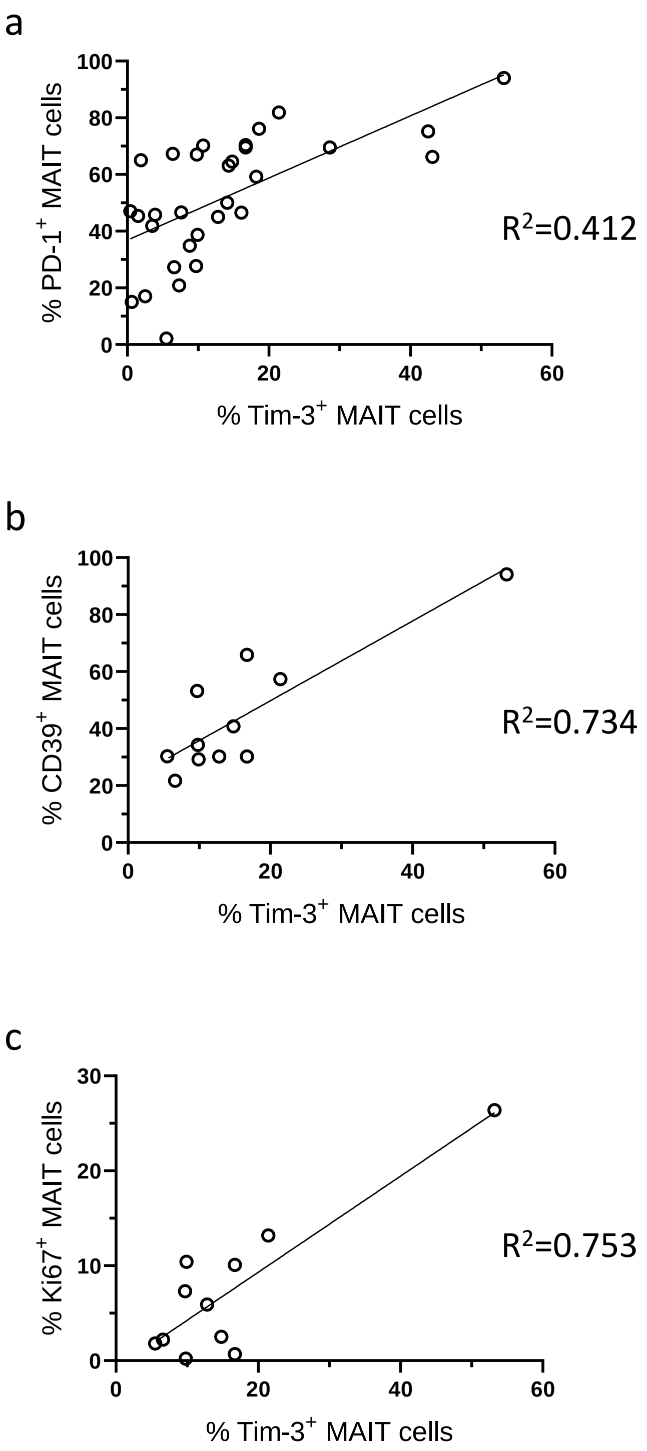

Supplementary Fig. S3. Correlation between exhaustion markers in intratumoral MAIT cells. Single cell suspensions were isolated from colon tumors, and expression of Tim-3, PD-1, CD39, and Ki67 by $CD8^+$ MAIT cells evaluated by flow cytometry. Graphs show correlation between percentage of $Tim\text{-}3^+$ cells and percentage of (a) $PD\text{-}1^+$, (b) $CD39^+$, and (c) $Ki67^+$ cells using linear regression. n=11-31

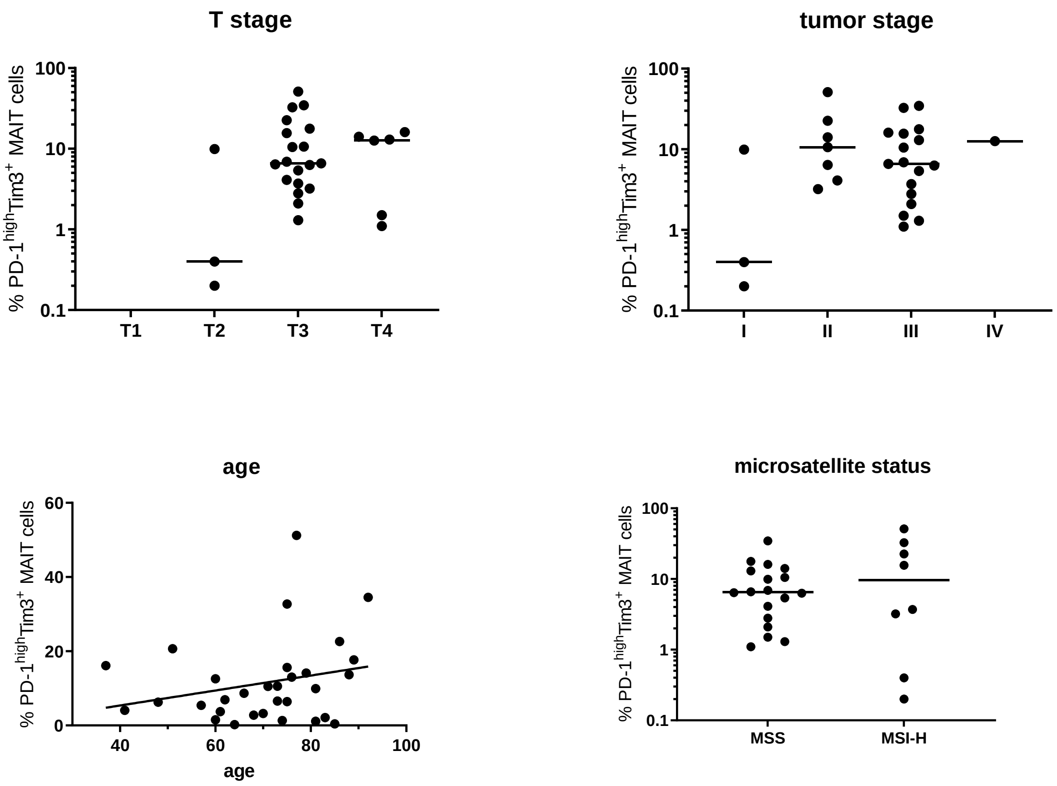

Supplementary Fig. S4 Frequencies of exhausted MAIT cells in relation to patient and tumor characteristics. Single cell suspensions were prepared from colon tumors, and frequencies of $CD8^+$ MAIT cells co-expressing PD-1 and Tim-3 determined by flow cytometry in freshly isolated cells. n=26-31

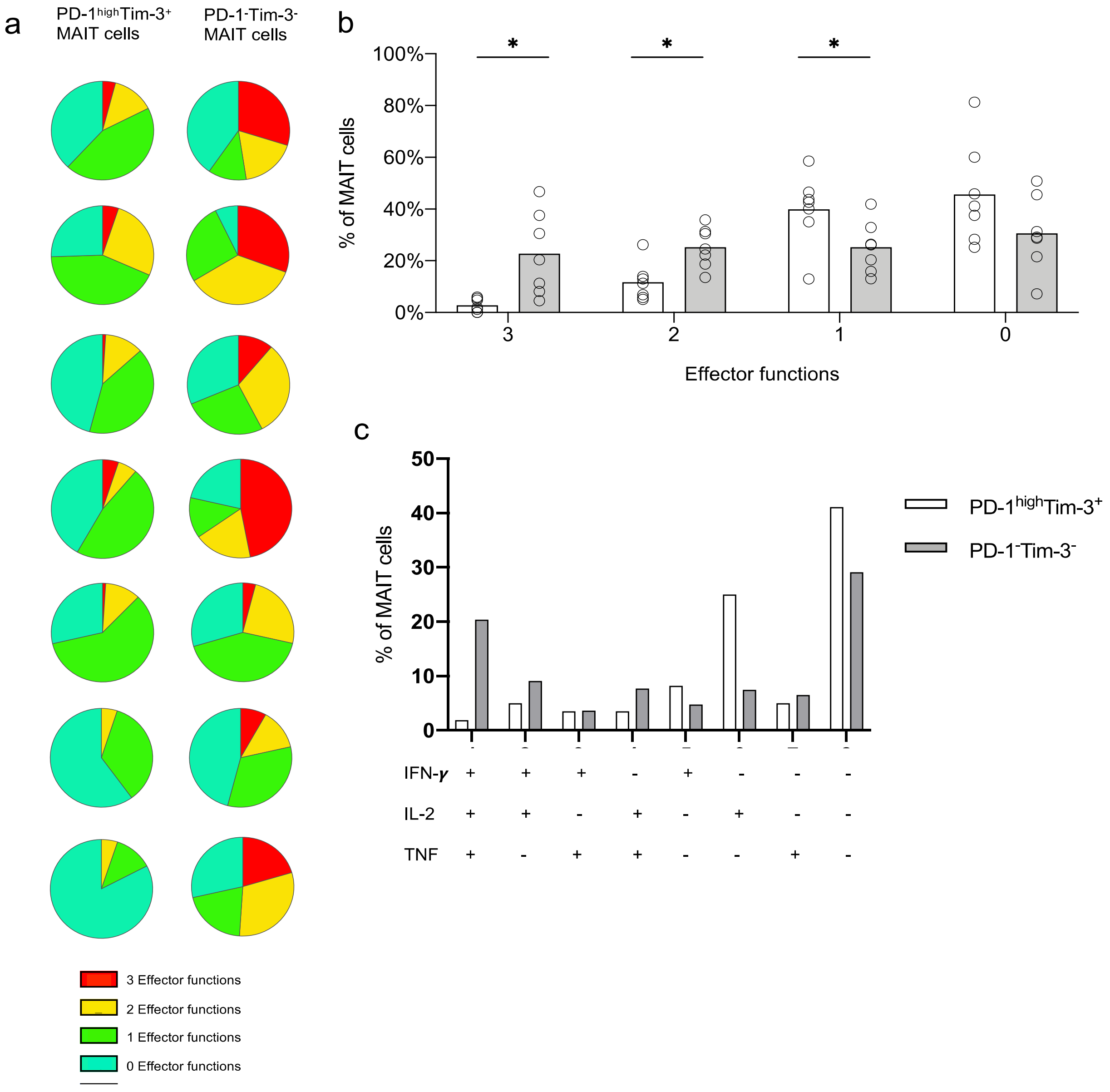

Supplementary Fig. S5 Polyfunctionality in tumor-infiltrating MAIT cells. Single cell suspensions were isolated from colon tumors, and production of IFN-γ, IL-2, and TNF by MAIT cells evaluated in vitro by flow cytometry after polyclonal stimulation with PMA and Ionomycin. (a) Percentage of PD-1^{hi}Tim-3^{+} and PD-1^{-}Tim-3^{-} MAIT cells from individual patients expressing 3 (red), 2 (yellow), 1 (green), or 0 (turquoise) of the analyzed cytokines illustrated as pie charts. (b) Frequencies of PD-1^{hi}Tim-3^{+} (white bars) and PD-1^{-}Tim-3^{-} (grey bars) MAIT cells expressing 3, 2, 1 or 0 of the analyzed effector molecules, shown as the percentage of total MAIT cells. (c) Frequencies of PD-1^{hi}Tim-3^{+} (white bars) and PD-1^{-}Tim-3^{-} (grey bars) MAIT cell subsets expressing a combination of IFN-γ, IL-2, and TNF shown as the percentage of total MAIT cells. Symbols represent individual values and bars the mean. *<0.05 using Wilcoxon matched-pairs signed rank test, n=7p

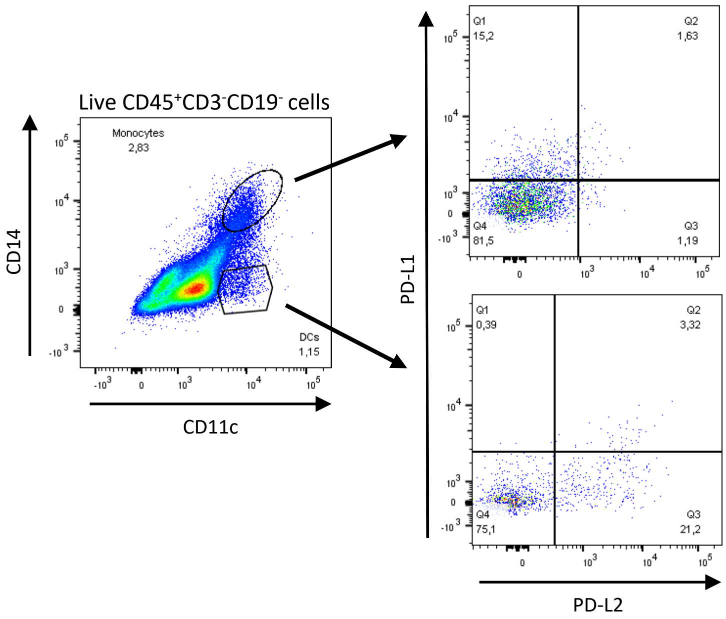

Supplementary Fig. S6 Representative flow cytometry analysis showing the expression of PD-L1 and PD-L2 among putative monocytes/immature macrophages ($CD14^{+}CD11c^{+}$) and dendritic cells ($CD14^{-}CD11c^{+}$) gated from live $CD45^{+}CD3^{-}CD19^{-}$ single cells isolated from a colon tumor. Gates are set according to FMO controls gated from the respective cell suspensions.

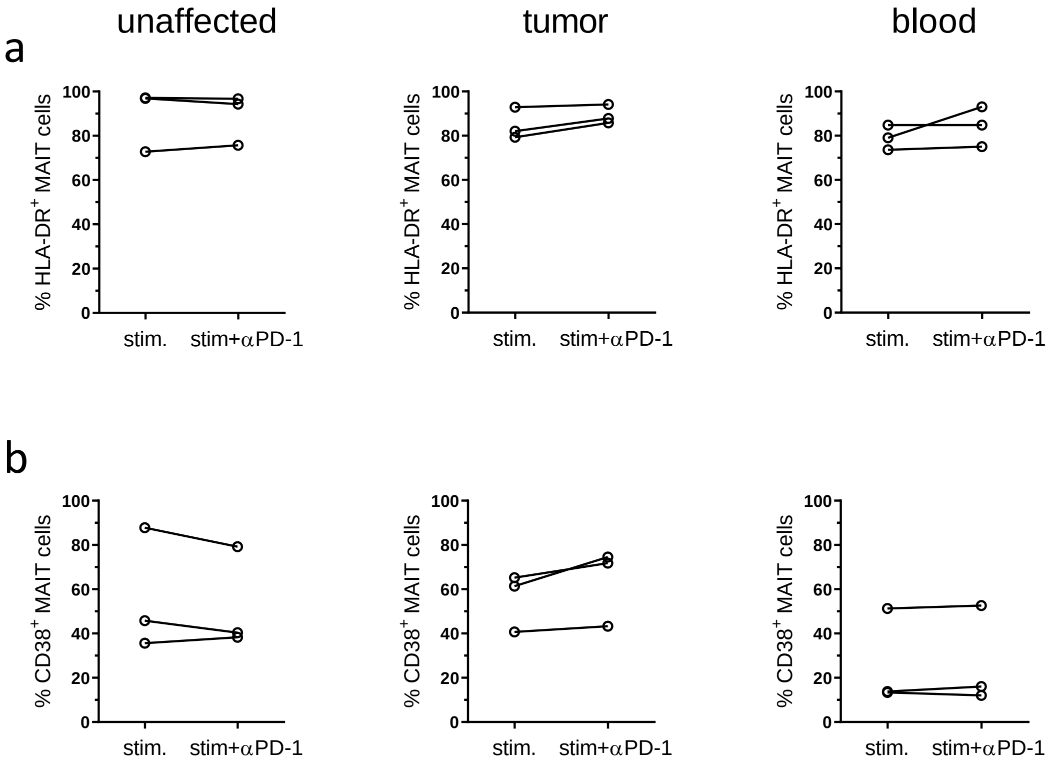

Supplementary Fig. 7 MAIT cell activation after PD-1 blocking. Single cell suspensions were isolated from unaffected colon, colon tumors and peripheral blood, and stimulated with THP-1 cells pre-incubated with *E. coli* in the presence or absence of blocking antibodies to PD-1. MAIT cell expression of HLA-DR (a) and CD38 (b) was evaluated by flow cytometry. Symbols represent individual values, and are connected to show corresponding values in the same individuals.
