# Supplementary material for: Exhaustion in tumor-infiltrating Mucosal-Associated Invariant T (MAIT) cells from colon cancer patients
Source: Cancer Immunol Immunother. 2021 Apr 22;70(12):3461–75. doi: 10.1007/s00262-021-02939-y (PMC8571139; doi:10.1007/s00262-021-02939-y)
Supplement: Supplementary file 2 — Supplementary file2 (PDF 104 kb) [file 262_2021_2939_MOESM2_ESM.pdf]

Supplementary table I. Characteristics of the colon cancer patients included in the study.

		females	males
	n	19	28
	age	41-92	37-88
Tumor location	ceacum	4	5
	ascending	9	4
	transverse	4	7
	descending	-	3
	sigmoid	2	9
TNM stage	I	1	3
	II	8	9
	III	10	16
	IV	-	1
Microsatellite status	MSS	7	18
	MSI-H	10	4
	unknown	2	6
